# Bibliometric analysis of nurses’ moral distress research

**DOI:** 10.3389/fpsyt.2024.1504713

**Published:** 2024-12-24

**Authors:** Xin Li, Xiao Liu, Fengxia Wang, Yi Zhang, Jianxin Huang, Jihong Wang, Xiaohuan Zhou

**Affiliations:** ^1^ School of Nursing, Pingdingshan University, Pingdingshan, Henan Province, China; ^2^ Department of Nursing, Hubei University of Medicine, Shiyan, Hubei Province, China

**Keywords:** moral distress, nurses, moral resilience, burnout, bibliometric analysis, research trends, research hotspots

## Abstract

**Objective:**

To identify the research status of nurses’ moral distress and predict emerging research hotspots and development trends.

**Methods:**

Articles on nurses’ moral distress were retrieved from the Web of Science Core Collection database from the inception of the database to 2024. A bibliometric analysis was conducted using VOSviewer and CiteSpace software to analyze publication distributions by country, institution, journal, author contributions, keyword trends, and reference co-citations.

**Results:**

Our study analyzed 1,781 documents, revealing a notable increase in publications after 2017, with contributions from 88 countries and 2,301 institutions worldwide. The United States and China were prominent contributors, highlighting global interest in this area. Analyses of keywords and cited references reveal emerging research topics such as “COVID-19”, “burnout”, and “moral resilience”.

**Conclusion:**

This bibliometric review sheds light on the growing academic interest in nurses’ moral distress, emphasizing key themes and outlining future research directions. By charting the development of this domain, our study provides critical insights, guiding the investigation of complex ethical issues in nursing and enhancing understanding of nurses’ moral distress.

## Introduction

Health professionals frequently encounter complex ethical dilemmas, often finding themselves constrained by circumstances that prevent them from acting following their beliefs and values (1). This conflict leads to frustration and powerlessness, commonly referred to as “moral distress” (2). Nurses, as primary caregivers intimately involved in patient care, are particularly susceptible to moral distress due to their critical role in ethically charged situations (3).

Studies from various regions have highlighted the global prevalence of moral distress. In the United States, approximately one in three nurses reported experiencing moral distress in their professional roles (4). In Ethiopia, Berhie et al. (5) observed that 83.7% of 423 ICU nurses were affected by moral distress. Similarly, Schulz et al. (6) found that 68% of 281 nurses at a tertiary pediatric center in Australia faced this issue.

Moral distress often arises in the inherently stressful hospital environment. Ethical challenges include inadequate end-of-life care (7), intra-team conflicts (8), and administrative issues such as misaligned policies, insufficient patient-to-staff ratios, resource shortages, and poor ethical climates exacerbated by inadequate departmental support (9, 10).

Unresolved moral distress significantly impacts both nurses and the quality of care they provide. It compromises patient safety, satisfaction, and care outcomes (11, 12). Additionally, nurses frequently experience helplessness and self-denial, posing substantial risks to their mental health (13, 14). Furthermore, it is also linked to increased stress, compassion fatigue, job burnout, turnover intention, and decreased work engagement (15–17). Recognizing its pervasive impact, the American Nurses Association (ANA) (18) and the American Association of Critical Care Nurses (AACN) (19) have called for greater awareness and interventions to address moral distress in healthcare settings.

Despite the growing attention to moral distress among nurses as a critical research area, bibliometric studies on this topic remain scarce. Bibliometric analysis, which involves the quantitative examination of research publications using mathematical and statistical methods, offers valuable insights into the development of scientific literature (20, 21). Tools like VOSviewer and CiteSpace enable researchers to conduct visual analyses of global trends and key areas of focus in the literature (22, 23). The Web of Science (WOS) database, widely regarded for its accessibility and reliability, serves as a primary resource for bibliometric analysis (24–26).

In this paper, we utilize VOSviewer and CiteSpace to analyze publications related to moral distress among nurses, focusing on the number of papers, authors, journals, countries, institutions, and keywords within this field. Our study aims to assess the current state of research on nurses’ moral distress and to identify emerging hotspots and future trends in this important area of healthcare.

## Methods

### Study design

This study employed a bibliometric analysis to investigate publications related to nurses’ moral distress. The analysis encompassed publications sourced from the Web of Science Core Collection database, spanning from its inception to 2024.

### Data collection and search strategy

**Figure 1:**
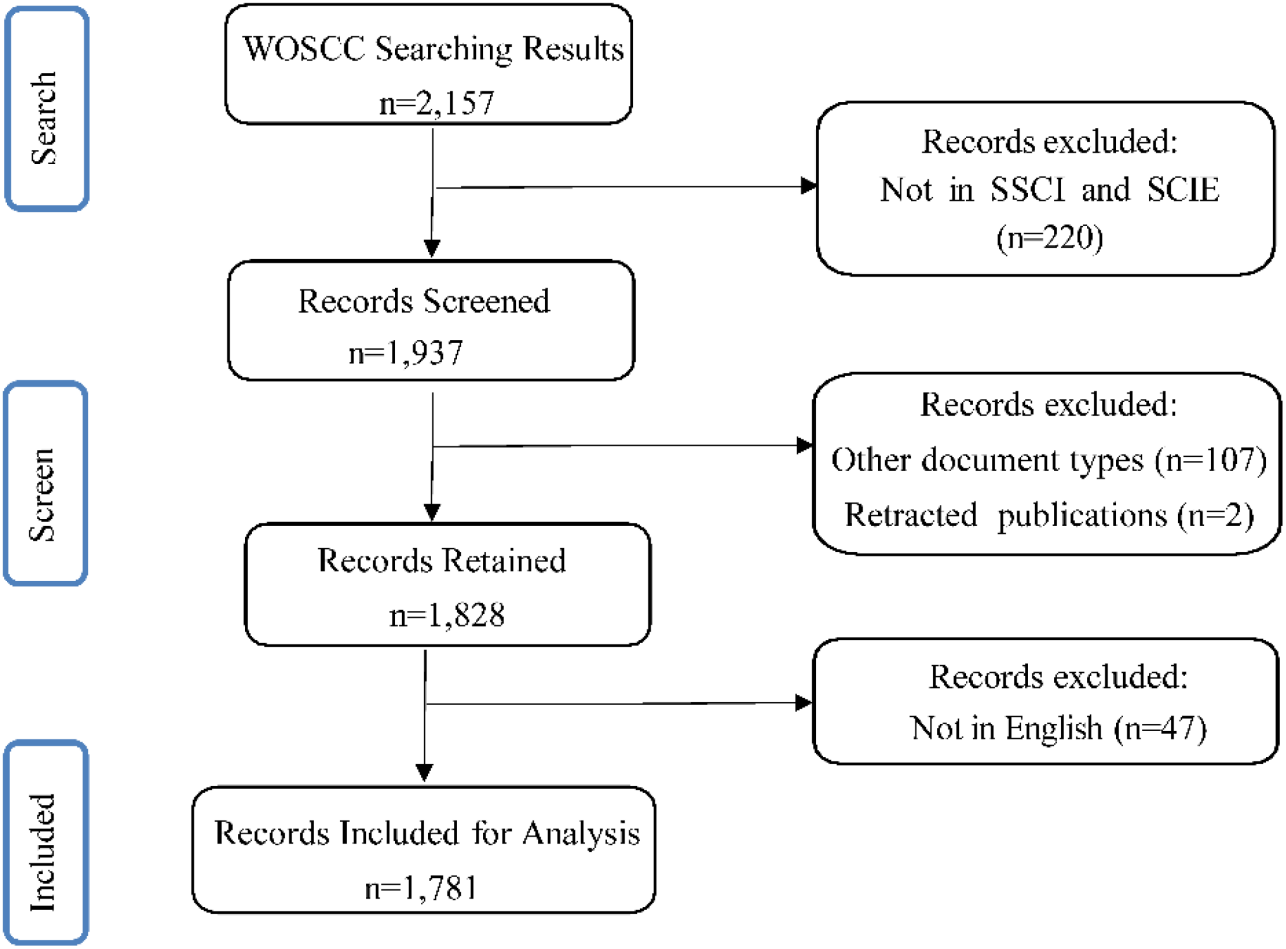
Flowchart of the literature selection process. Other document types include Editorial Materials, Meeting Abstracts, Proceeding Papers, Letters, Book Reviews, and Corrections.

The publications for this study were sourced from the Web of Science Core Collection (WOSCC) on Feb 14, 2024. The retrieval formula was as follows: Topic (TS)=(“moral distress” or “moral dilemma*” or “ethical dilemma*” or “ethical conflict*” or “moral tension” or “moral uncertainty” or “moral constraint*” or “moral conflict*”) AND Topic (TS)=(“nursing assistant*” OR “nursing personnel” OR “nursing professional*” OR “nursing provider*” OR “nursing staff” OR “nursing worker*” OR “nursing practitioner*” OR “nurse” OR “nurses” OR “nurse specialist*” OR “nurse practitioner*”). The literature search spanned from the inception of the databases to 2024. The search results were refined by the Science Citation Index-Expanded (SCIE) and the Social Sciences Citation Index (SSCI). Editorial Materials, Meeting Abstracts, Proceeding Papers, Letters, Book Reviews, Corrections, and Retracted Publications were excluded from the analysis. Only Articles and Reviews published in English were included. This yielded 1,781 relevant records for inclusion in the analysis, as illustrated in **Figure 1**. Data were extracted from the WOSCC in TXT format. To ensure methodological rigor, two independent researchers conducted the literature review and data analysis, with a third researcher resolving any discrepancies. A systematic approach was employed to identify and integrate synonyms and near-synonyms, ensuring a thorough and accurate analysis.

### Data analysis and visualization methods

The study employed CiteSpace 6.2. R2 (Chaomei Chen, Drexel University, USA), VOSviewer 1.6.1 (Centre for Science and Technology Studies, Leiden University, the Netherlands), Scimago Graphica 1.0.34 (International University of la Rioja, Spain), and Microsoft Excel 2019 (Microsoft Corporation, United States) as analysis tools. Cooperative co-occurrence graphs, illustrating contributions from various countries, authors, and institutions, were used to analyze the connections among these elements. Cluster analysis (27) and keyword burst detection were conducted to trace the evolution of research hotspots and predict emerging trends. Dual-map overlays were utilized to reveal the relationships between cited and citing journals, enhancing the analysis of disciplinary literature. Co-citation analysis helped identify classical literature within the field. A world map was generated by Scimago Graphica to visually depict the global distribution of research on moral distress among nurses. Data processing was conducted using the WOSCC (Clarivate Analytics) and Microsoft Excel 2019, with Journal Impact Factors sourced from the 2022 Journal Citation Reports (Clarivate Analytics).

## Results

### Analysis of annual publications and trends

**Figure 2:**
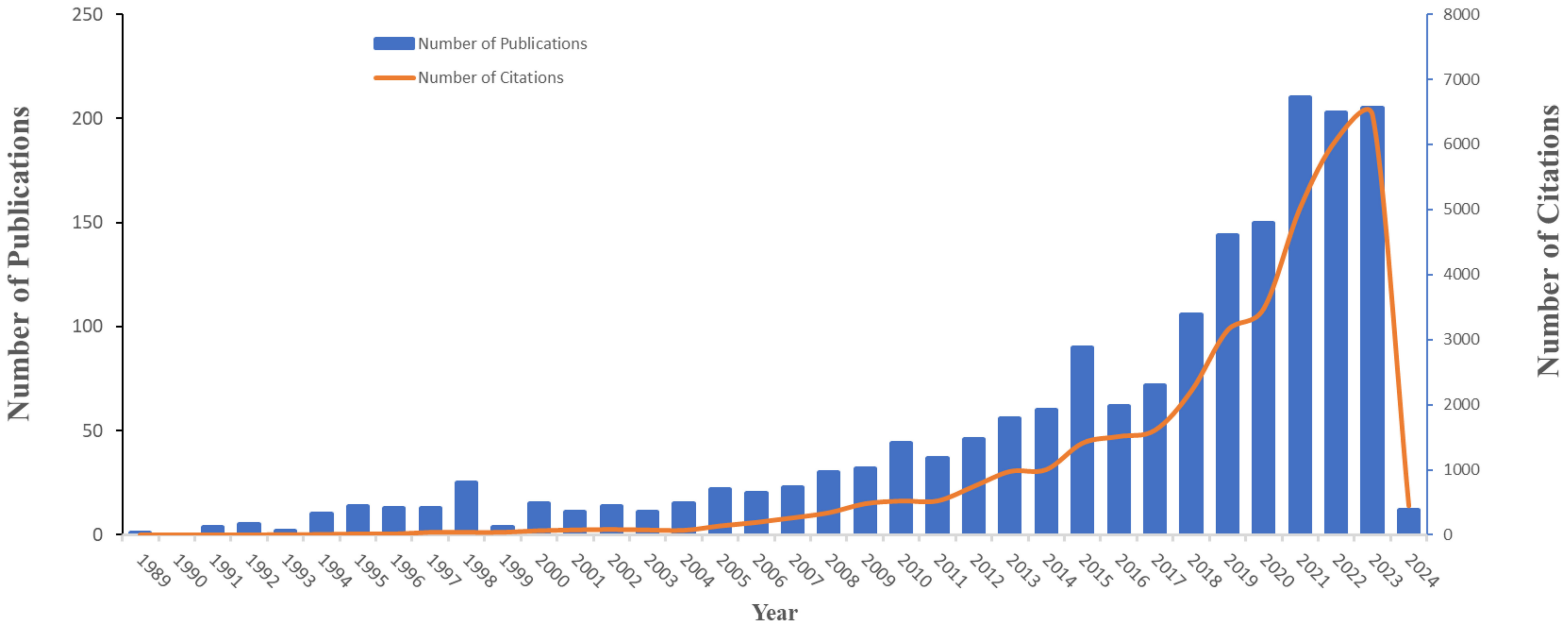
Trends in annual publications and citations on nurses’ moral distress worldwide from 1989 to 2024.

**Figure 2** presents the chronological trends in research on moral distress among nurses, beginning with the pioneering study in 1989 titled “Moral Distress and the Shortage of Critical Care Nurses.” The publication trend was divided into two distinct phases. From 1989 to 2017, the annual number of publications was relatively low, seldom surpassing 100 articles. However, a significant surge occurred afterward, with the number of publications peaking at 210 articles in 2021. In total, these studies achieved 37,124 citations, with an average of 20.84 citations per article, highlighting the increasing academic interest and engagement in this field.

### Countries and institutions

**Figure 3:**
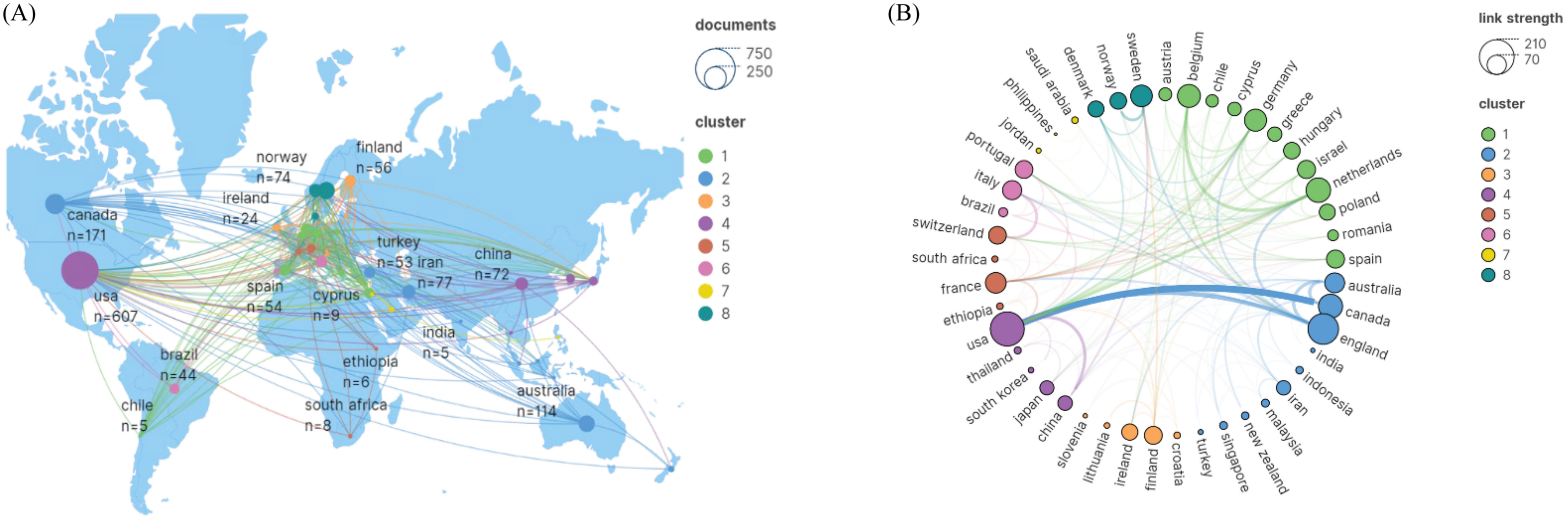
The collaborative dynamics between nations in nurse-related moral distress research. **(A)** The global geographical distribution of leading contributors. **(B)** An inter-country collaboration network in nurse-related moral distress research based on VOSviewer and Scimago.

**Figure 3A** presents an overview of global research on nurse-related moral distress, involving contributions from 88 countries/regions. The study encompasses 1,781 publications from 2,301 distinct institutions worldwide. The United States emerged as the leading contributor in this analysis, with 607 articles (34.08%) and 14,388 citations. Canada, England, Sweden, and Australia followed the United States as major contributors, with 171 (9.60%), 139 (7.80%), 123 (6.91%), and 114 (6.40%) publications respectively. The top 10 countries collectively accounted for 1,490 of the 1,781 articles, detailed in **Table 1**. Additionally, **Figure 3B** depicts the patterns of international collaboration, emphasizing the United States’ central role. Notably, extensive collaboration occurred among the United States, England, Canada, and China.

**Table 1:** The top 10 prolific countries and institutions of publications on moral distress among nurses.

Rank	Countries	Publications	Citations	Institution	Publications	Citations
1	USA	607	14388	Karolinska Institutet	32	437
2	Canada	171	3863	University of Alberta	30	624
3	England	139	3350	University of Toronto	29	542
4	Sweden	123	2755	University of Turku	28	737
5	Australia	114	2329	University of Washington	22	356
6	Iran	77	1142	University of Pennsylvania	22	650
7	Norway	74	1401	The University of British Columbia	21	786
8	China	72	1027	The University of Virginia	21	1148
9	Netherlands	57	171770	University of Oslo	19	618
10	Italy	56	1315	Johns Hopkins University	18	811

**Figure 4:**
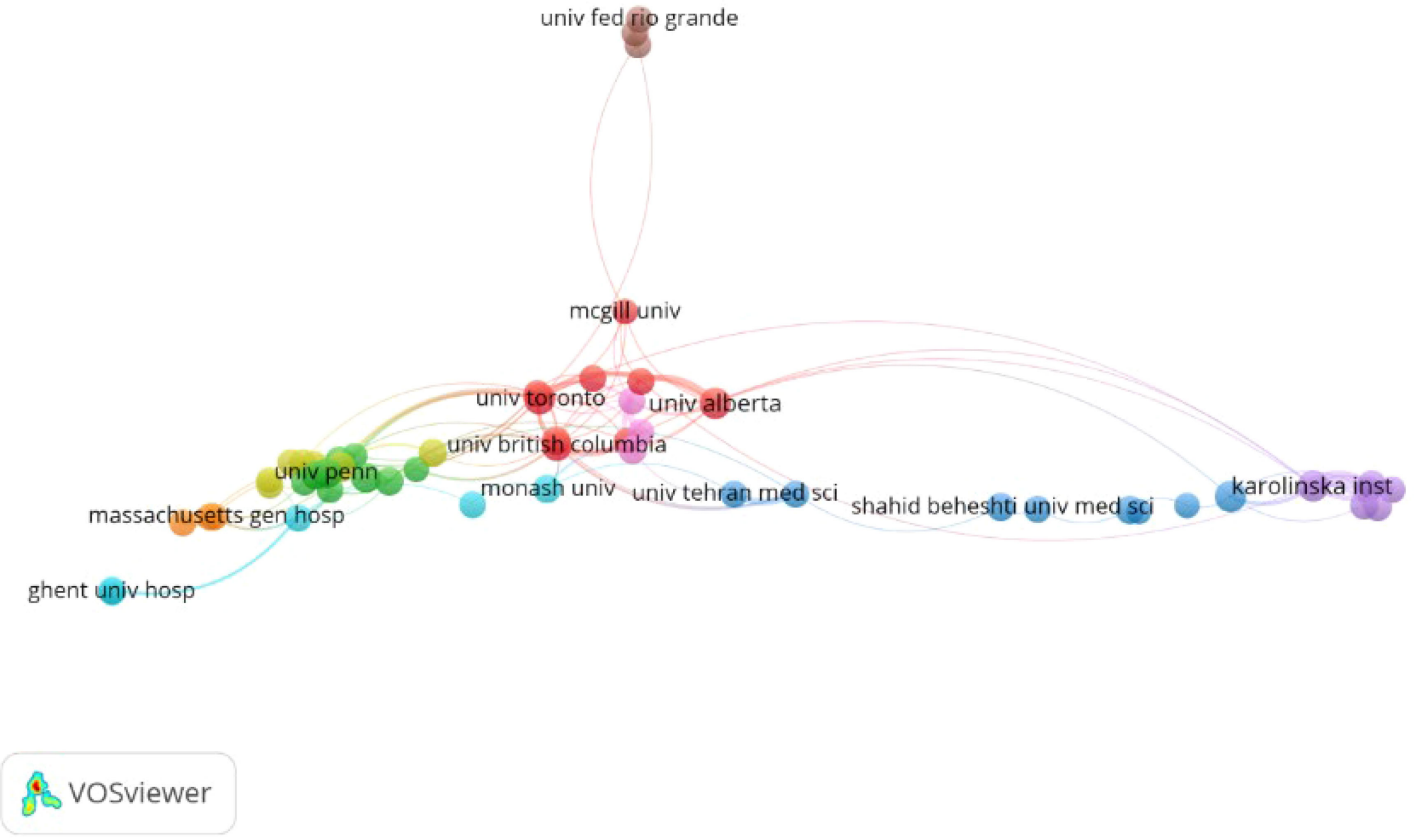
Collaboration network of institutions in moral distress among nurses research based on VOSviewer.

The leading research institutions in terms of productivity included Karolinska Institutet with 32 publications (1.80%), the University of Alberta with 30 publications (1.68%), the University of Toronto with 29 publications (1.63%), and the University of Turku with 28 publications (1.57%), as detailed in **Table 1**. The institutional collaboration network revealed extensive cooperation among the University of Toronto, the University of Washington, the University of Alberta, the University of British Columbia, and the Karolinska Institutet, as illustrated in **Figure 4**.

### Analysis of authors and co-cited authors

Our study identified 5,711 scholars dedicated to researching nurses’ moral distress, with the majority (97.37%) authoring only one to three articles. The top 10 most prolific and frequently co-cited authors are listed in **Table 2**. Rushton, C.H. led with 15 publications (28–42), closely followed by Devos Barlem, E.L. and Dalmolin, G., each with 13 contributions. In terms of co-citations, Corley, M.C. emerged as the most cited author with 745 citations, followed by Hamric, A.B. with 630 and Jameton, A. with 599 citations, underscoring their pivotal contributions to nurse-related moral distress research, as detailed in **Table 2**.

**Table 2:** The top 10 productive and frequently co-cited authors on moral distress among nurses.

Rank	Author	Publications	Co-cited author	Citations
1	Rushton, C.H.	15	Corley, M.C.	745
2	Devos Barlem, E.L.	13	Hamric, A.B.	630
3	Dalmolin, G.	13	Jameton, A.	599
4	Leino-Kilpi, H.	13	Rushton, C.H.	356
5	Falco-Pegueroles, A.	12	Epstein, E.G.	273
6	Suhonen, R.	11	Morley, G.	217
7	Morley, G.	11	McCarthy, J.	199
8	Ergert, P.	10	Austin, W.	175
9	Brown-Saltzman, K.	10	Wilkinson, J.M.	171
10	Kim, S.	10	Lamiani, G.	167

### Journals and co-cited journals

The analysis included 383 journals. **Table 3** lists the top 10 journals that have made significant contributions to the field of nurses’ moral distress. *Nursing Ethics* (IF=4.2) was the leading journal in the field of nurses’ moral distress, publishing 405 papers, which accounted for nearly a quarter of the total output in this field. These publications received 1,972 citations, with an average of 25.0 citations per paper. The *Journal of Advanced Nursing* (IF=3.8) followed with 63 publications, while the *Journal of Clinical Nursing* (IF=4.2) published 43 articles. Most of the prominent journals were based in England. Among them, the *International Journal of Nursing Studies* was particularly noteworthy for its high impact factor, with its articles receiving an average of 51.6 citations each.

**Table 3:** The top 10 prolific journals on moral distress among nurses.

Rank	Journals	Country	Publications	Citations	Average Citations	JCR	IF (2022)
1	Nursing Ethics	England	405	10,126	25.0	Q1	4.2
2	Journal of Advanced Nursing	England	63	2,564	40.7	Q1	3.8
3	Journal of Clinical Nursing	England	43	1,411	32.8	Q1	4.2
4	Journal of Nursing Management	England	31	574	18.5	Q1	5.5
5	International Journal of Environmental Research and Public Health	Switzerland	31	294	9.5	/	/
6	BMC Nursing	England	31	193	6.2	Q1	3.2
7	BMC Medical Ethics	England	30	403	13.4	Q1	2.7
8	Journal of Hospice & Palliative Nursing	USA	30	139	4.6	Q3	1.8
9	International Journal of Nursing Studies	England	26	1,342	51.6	Q1	8.1
10	Journal of Nursing Administration	USA	26	176	6.8	Q2	2.0

JCR, journal citation reports; IF, impact factor; Q1, Q2, Q3, Quartile rankings in the Journal Citation Reports, with Q1 representing the top 25% of journals in a specific field, Q2 representing the top 25%-50%, and Q3 representing the top 50%-75%.

**Figure 5:**
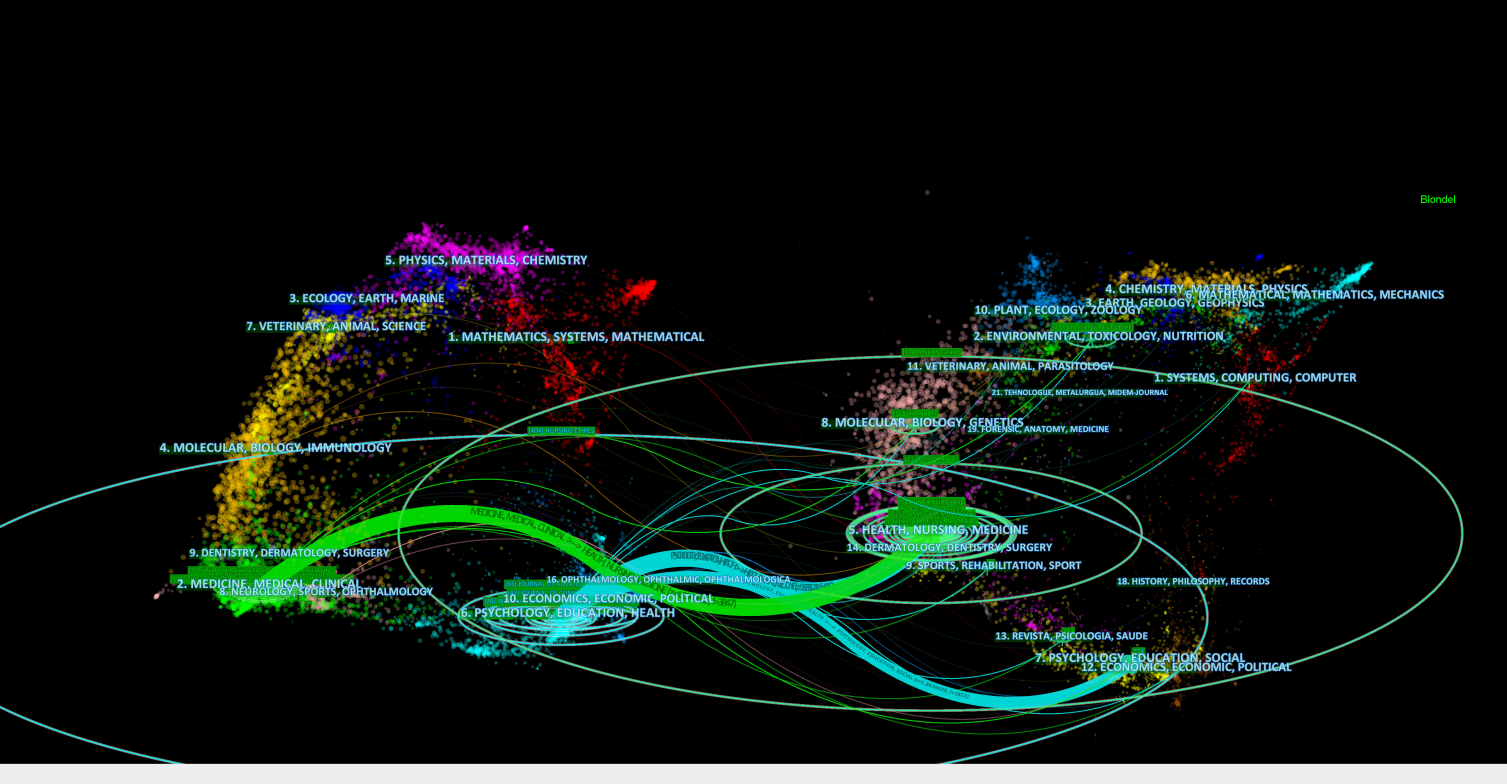
Dual-map overlay of citing and cited journals in nurses’ moral distress. The figure’s left panel illustrates the distribution of journals citing this research, and the right panel shows the journals being cited. Lines linking the two panels signify citation connections, with accompanying labels identifying the subjects covered. Colored pathways demonstrate the citation flow from the left-side citing literature to the right-side cited literature.

The dual-map overlay technique, which juxtaposes journals and disciplines, provides a detailed depiction of the dissemination of scholarly journals across various fields, traces citation trajectories, and emphasizes changing focuses in scientific research (43). The dual-map overlay of the journal identified three main citation pathways (**Figure 5**). Articles published in the Health/Nursing/Medicine disciplines were often cited by articles published in the Medicine/Medical/Clinical and Psychology/Education/Health. However, articles published in the Psychology/Education/Social disciplines were mostly cited by articles published in Psychology/Education/Health.

### Co-cited references

Co-cited references analysis was employed to explore the simultaneous citation of two documents within the reference list of a third publication. **Table 4** lists the top 10 most cited articles on nurses’ moral distress. Based on the most cited source, the 27-item revised Measure of Moral Distress for Healthcare Professionals scale (44), designed to encompass the most extensively recognized causes of moral distress, was developed and validated for evaluating moral distress in healthcare professionals. The second most cited paper described the moral distress among all healthcare professionals and all settings in one large healthcare system (45). Among the top ten co-cited papers, four were published in *Nursing Ethics*.

**Table 4:** The top 10 highly cited references on moral distress among nurses.

Rank	Reference Title	First author	Journal	Year	Citation count	
1	Enhancing Understanding of Moral Distress: The Measure of Moral Distress for Health Care Professionals	Epstein Elizabeth G	AJOB Empirical Bioethics	2019	104
2	Moral Distress Among Healthcare Professionals: Report of an Institution-Wide Survey	Whitehead PB	Journal of Nursing Scholarship	2015	65
3	When healthcare professionals cannot do the right thing: A systematic review of moral distress and its correlates	Lamiani G	Journal of Health Psychology	2017	65
4	Moral distress in critical care nursing: The state of the science	McAndrew NS	Nursing Ethics	2018	58
5	Moral distress experienced by nurses: A quantitative literature review	Oh Y	Nursing Ethics	2015	55
6	What is ‘moral distress’? A narrative synthesis of the literature	Morley G	Nursing Ethics	2019	47
7	Moral distress and its contribution to the development of burnout syndrome among critical care providers	Fumis RRL	Annals of Intensive Care	2017	44
8	Moral distress in intensive care unit professionals is associated with profession, age, and years of experience	Dodek PM	Journal of Critical Care	2016	40
9	A Health System-wide Moral Distress Consultation Service: Development and Evaluation	Hamric AB	HEC Forum	2017	39
10	Registered Nurses’ Perceptions of Moral Distress and Ethical Climate	Pauly B	Nursing Ethics	2009	38

### Keyword analysis

**Figure 6:**
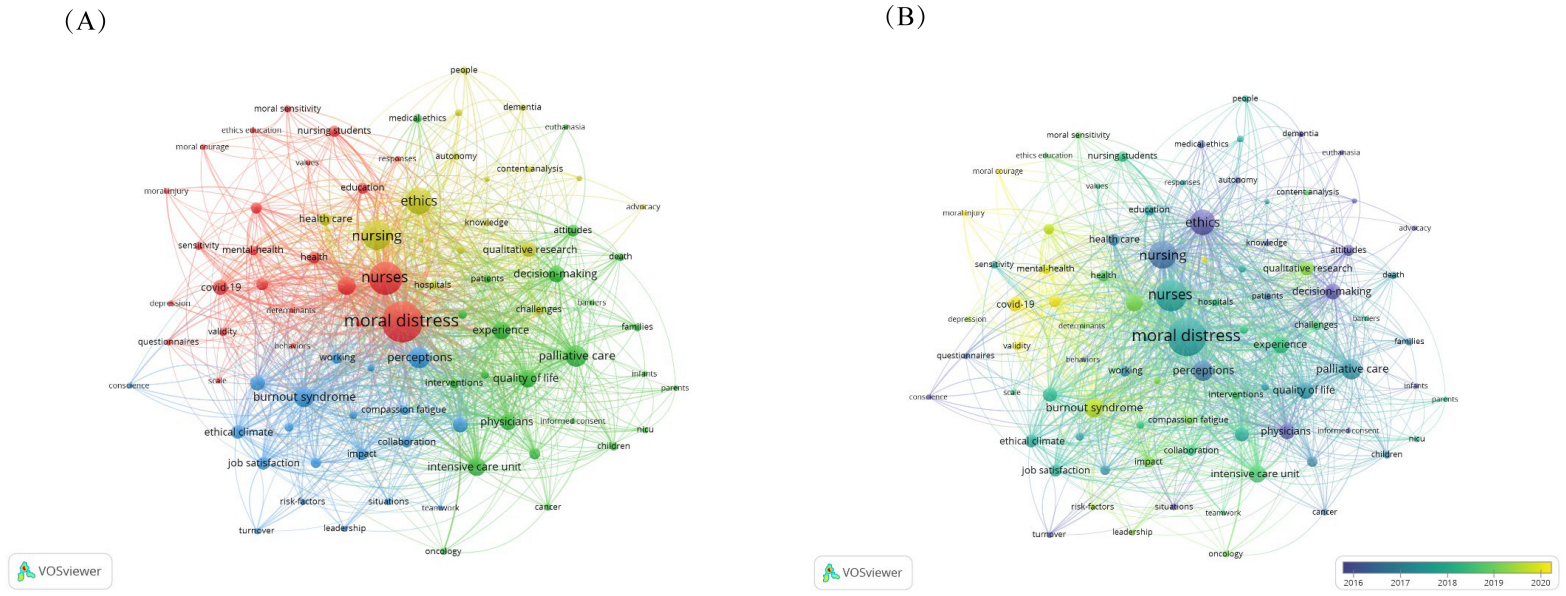
VOSviewer keywords co-occurrence view. **(A)** Keywords clustering visualization; **(B)** Keywords co-occurrence overlay visualization.

Keywords represent the core of an article, succinctly capturing its primary content. Frequently occurring keywords often highlight emerging trends and define the research frontier. Eighty-five keywords with a minimum of 20 occurrences were determined using VOSviewer keyword analysis of the 1,781 articles (**Figure 6**). Clusters were grouped according to commonly used keywords by researchers and were presented using different colors. As shown in **Figure 6A**, the 85 keywords were divided into four clusters. Cluster 1 had 24 keywords mainly related to mental health, COVID-19, and moral resilience. Cluster 2, with 24 keywords, focused on experience, quality of life, palliative care, and intensive care. Cluster 3 included 20 keywords centered on stress, burnout, and job satisfaction. Cluster 4 comprised 17 keywords focused on qualitative research, with strong links between the clusters.

**Figure 6B** depicts the temporal evolution of keywords. Keywords represented in purple denote earlier appearances, whereas those in yellow indicate more recent introductions. In recent years, keywords such as “COVID-19”, “burnout”, “moral resilience”, and “mental health” have emerged prominently.

**Figure 7:**
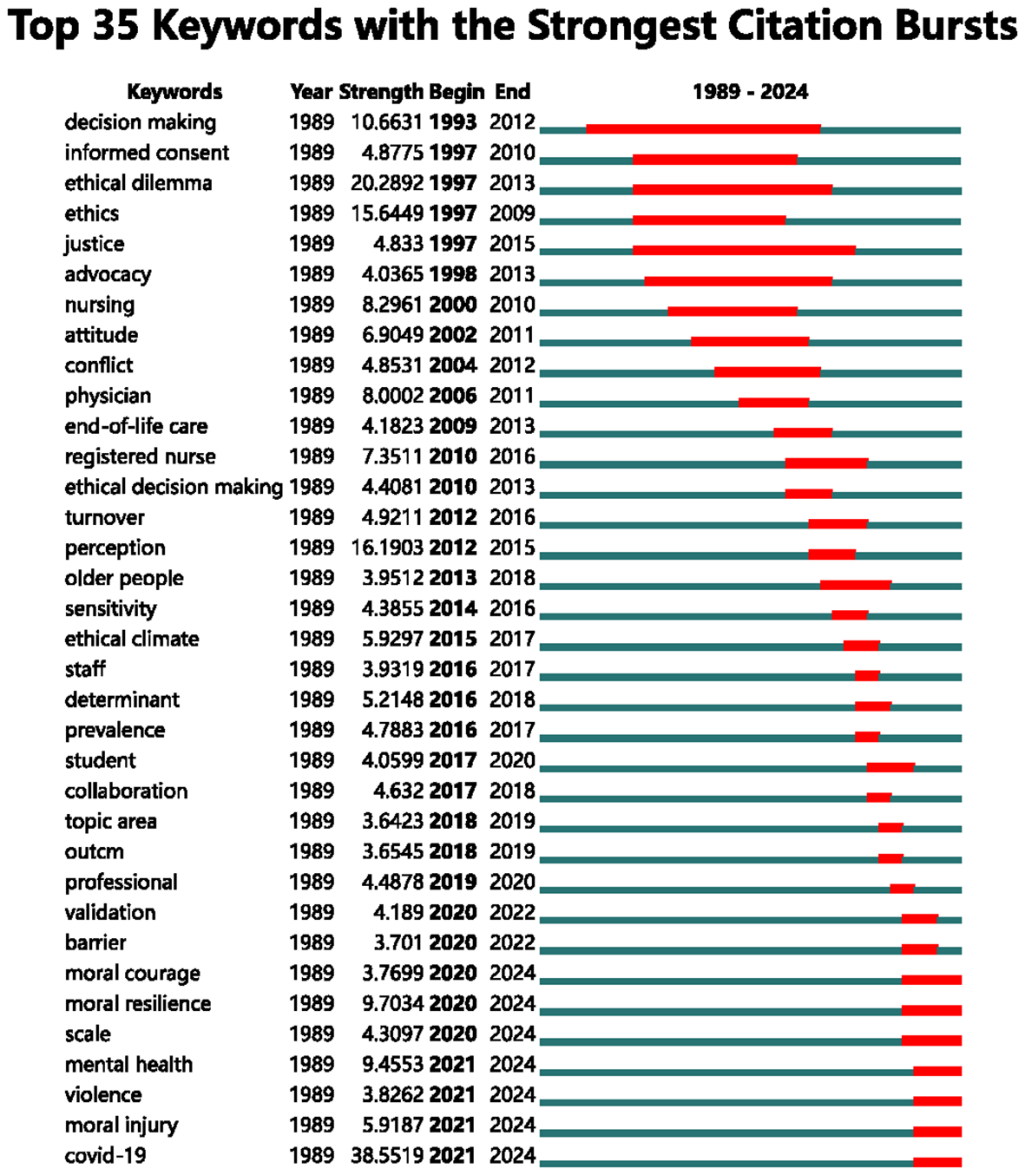
Top 35 keywords with the strongest citation bursts.

Analyzing burst keywords facilitates the identification of research hotspots in nurses’ moral distress across various stages. **Figure 7** reveals that from 1993 to 2015, “ethical dilemma”, “perception”, and “ethics” emerged as the predominant keywords in research on nurses’ moral distress, suggesting a focus on their moral distress and perceptions. Since 2020, “COVID-19”, “moral resilience”, and “mental health” have been the most frequently occurring keywords in studies on nurses’ moral distress, reflecting a recent emphasis on the effects of COVID-19, moral resilience, and mental well-being of nurses.

## Discussion

This study provides the first bibliometric analysis spanning over 30 years of research on moral distress among nurses. The analysis revealed an overall increase in the number of published articles, divided into two distinct phases. The first phase, from 1989 to 2017, was marked by a relatively low number of publications each year, possibly due to limited attention to this issue within the nursing community and restricted funding. The second phase, from 2018 to 2023, saw a significant rise in publications focused on nurses’ moral distress. This increase was largely attributed to heightened awareness and strategic efforts by organizations such as ANA to address these ethical challenges. Furthermore, the surge in research during this period has been linked to the profound impact of the COVID-19 pandemic, which brought unprecedented ethical challenges to the forefront of healthcare. The growing interest in nurses’ moral distress has driven continuous scholarly attention to this critical topic.

This study indicated that among 88 countries, the United States was the leading contributor to publications on nurses’ moral distress. This finding contrasts with a previous systematic review by Alimoradi et al. (46), which identified Iran as the leading country in terms of eligible publications. The discrepancy arose primarily from the earlier review’s exclusive focus on observational studies, whereas our study employs broader inclusion criteria. The dissemination of research findings through leading international journals has had wide-reaching implications. Notably, since 2018, there has been a significant upward trend in research on nurses’ ethical distress. *Nursing Ethics* emerged as the foremost journal publishing research on this topic, closely followed by the *Journal of Advanced Nursing*.

The findings suggested that research on nurses’ moral distress was primarily concentrated within top institutions, predominantly located in the United States and Canada. While some contributions from Asian institutions were noted, the academic output and impact were largely driven by Western entities. Given Asia’s large patient population, increased contributions from this region could significantly influence the field.

Analysis of institutional collaboration networks indicated that collaborative research on nurses’ moral distress tended to center geographically. Notably, institutions like the University of Toronto and the University of Washington exhibited high levels of collaborative research and international partnerships. However, cross-border collaborations were less frequent compared to domestic partnerships. In Canada, the University of Toronto and the University of Alberta were particularly distinguished for their strong collaborative efforts and international connections. For instance, the University of Toronto collaborated extensively with national partners, such as the University of British Columbia and the University of Alberta, and with international institutions like the University of Washington in the United States. This network of collaborations highlighted the crucial role of institutional partnerships in advancing research and understanding of nurses’ moral distress.

This study analyzed the 10 most cited papers on moral distress, consisting of four reviews and six original studies, all employing quantitative methods. These highly cited references covered various research aspects of moral distress, including its prevalence, causes, effects, measurement techniques, and interventions. Notably, four of these key references originated from the United States, reflecting the country’s robust research infrastructure and the profound commitment to healthcare ethics.

The bibliometric analysis highlighted “burnout”, “moral resilience”, and “COVID-19” as focal areas of recent scholarly interest (**Figure 6B**). Burnout, recognized as a significant occupational disorder, is characterized by emotional exhaustion, depersonalization, and reduced personal accomplishment. Research indicated that nurses experience higher levels of burnout compared to those in other professions (47, 48). A distinct positive correlation has been observed between burnout and mental distress within this group (49–51). Johnson-Coyle et al. (52) posited that burnout might be the most severe consequence of moral distress. Furthermore, Xue et al. emphasized the crucial role of psychological capital in mediating the relationship between moral distress and burnout among nurses (51).

Moral resilience, a concept introduced to counteract moral suffering, represents the ability to maintain integrity and promote positive development in the face of ethical challenges (42, 53, 54). This emerging concept has shifted the focus from distress to empowerment, encouraging individuals to harness personal strengths in navigating moral dilemmas. Brewer et al. (55) identified modest but significant relationships between moral resilience and factors such as burnout, secondary traumatic stress, and compassion satisfaction. Chen et al. (56) investigated distinct latent profiles of moral resilience among registered nurses and examined how these profiles relate to compassion fatigue. Tian et al. (57) translated the Rushton Moral Resilience Scale (RMRS) into Chinese, enhancing its cross-cultural applicability and advancing the global understanding of moral resilience in healthcare.

Crisis care standards, grounded in a utilitarian ethical framework, often sharply contrast with the virtue-based ethical principles that typically guide nursing practice. The shift in clinical practices brought on by COVID-19 introduced novel ethical challenges, significantly increasing the likelihood of moral distress among healthcare professionals (58–60). This situation heightened the tension between making decisions for the greater good during a crisis and the traditional nursing focus on individual patient well-being, thereby exacerbating ethical conflicts and the associated distress. A study by Lake and colleagues (59) examined the effects of the initial COVID-19 surge on nurses’ moral distress. The findings revealed that factors such as clear and effective leadership communication, fewer COVID-19 patient assignments, and adequate access to protective equipment significantly reduce moral distress among nurses.

### Limitations

Like other bibliometric studies, this one also faced some limitations: (1) Despite the WOS’ recognized comprehensiveness and reliability in bibliometrics, it might not cover all relevant literature and citations in this field, potentially limiting the scope of the study and omitting critical studies. The primary reason for not including additional databases, such as MEDLINE or CINAHL, was the limitations of the software tools we used, which are optimized for analyzing data from Web of Science, PubMed, or Scopus. Integrating data from multiple databases, each with distinct indexing systems and metadata formats, presents technical challenges that could complicate the analysis and potentially introduce inconsistencies. Future studies should consider expanding database selection to achieve a more comprehensive dataset. (2) This study’s focus on English-language publications may have overlooked significant research and insights into nurses’ moral distress from non-English-speaking areas. Future research requires a broader linguistic perspective to comprehensively understand the global dimensions of nurses’ moral distress. (3) The analysis involved manually combining synonyms and similar terms for authors and keywords, complicated by the large number of contributors. Some authors may have changed their names or been associated with several institutions. The risk of bias from synonyms and similar terms was unavoidable. (4) The bibliometric analysis’s quantitative focus might not adequately capture the qualitative dimensions of nurses’ moral distress research, including study motivations and findings’ implications. Future studies should strive to integrate qualitative assessments to provide a more holistic understanding of nurses’ moral distress.

## Conclusion

The bibliometric review of nurses’ moral distress provides a quantitative, objective framework to analyze its evolution and current trends in this field. Since the early studies in 1989, research volume has grown significantly, especially since 2018, highlighting the increasing focus on nurses’ ethical challenges. The United States leads in research contributions, followed by Canada, England, Sweden, and Australia, showcasing the issue’s global nature and cross-border collaboration. This analysis highlights keywords of moral distress, such as “COVID-19”, “burnout”, and “moral resilience”. By analyzing leading authors, co-citations, and keyword trends, this study maps out the knowledge landscape of the field, identifying both established and emerging research themes. This comprehensive overview guides researchers by offering insights into potential research directions that can enhance our understanding and tackle nursing practice’s complex ethical challenges.
